# Rapidly progressive pulmonary abscess initially misdiagnosed as lung cancer: the role of *Peptostreptococcus stomatis* and *Parvimonas micra*

**DOI:** 10.3389/fcimb.2025.1590220

**Published:** 2025-06-11

**Authors:** Hang Hu, Kui Li, Zhidong Jin, Kaijin Wang, Bicui Liu

**Affiliations:** Bishan Hospital of Chongqing Medical University, Chongqing, China

**Keywords:** pulmonary abscess, immunocompetent patient, *Peptostreptococcus stomatis*, *Parvimonas micra*, rapidly progressive infection

## Abstract

Pulmonary abscess is a serious infectious disease characterized by localized lung tissue necrosis, primarily caused by anaerobic or facultative anaerobic bacterial infections. While the *Streptococcus anginosus group* (SAG) is a well-established pathogen in pulmonary abscess formation, recent findings suggest that strict anaerobes such as *Peptostreptococcus stomatis* and *Parvimonas micra* can also contribute to deep-seated infections. However, their role in rapidly progressive pulmonary abscesses has not been previously documented. Here, we present a case of a rapidly progressive pulmonary abscess caused by *Peptostreptococcus stomatis* and *Parvimonas micra* in an immunocompetent patient, initially misdiagnosed as lung cancer. This case highlights the importance of differentiating infectious lung lesions from malignancies and underscores the clinical utility of metagenomic next-generation sequencing (mNGS) in diagnosing rare anaerobic infections, offering valuable insights for precise diagnostic and therapeutic strategies.

## Introduction

Pulmonary abscess is a severe infectious condition characterized by localized lung tissue necrosis, most commonly resulting from anaerobic or facultative anaerobic bacterial infections (Sabbula et al., 2025). The *Streptococcus anginosus group* (SAG) is a well-recognized pathogen in pulmonary abscess formation (Kuhajda et al., 2015), but recent reports indicate that strict anaerobes such as *Peptostreptococcus stomatis* (Verdecia et al., 2019; Barnes et al., 2020; Hasel et al., 2022; Emmanuel et al., 2024) and *Parvimonas micra (* Yoo et al., 2019; Shimizu et al., 2022; Fukushima et al., 2023; Yang et al., 2023) may also play a role in deep-seated infections. These organisms are known for their strong suppurative potential, enabling them to invade various anatomical sites and cause severe infections, particularly in immunocompromised individuals. However, their association with rapidly progressive pulmonary abscesses in immunocompetent patients has not been previously described.

One of the key challenges in diagnosing pulmonary abscesses is their radiologic similarity to pulmonary malignancies. Computed tomography (CT) often reveals solitary lesions with irregular margins and central necrosis, features that closely resemble primary lung cancer or metastatic tumors. This overlap can lead to misdiagnosis, delays in appropriate treatment, and unnecessary invasive procedures.

Here, we present a rare case of a rapidly progressive pulmonary abscess caused by *Peptostreptococcus stomatis* and *Parvimonas micra*, which was initially mistaken for a malignant lung tumor. This case highlights the importance of distinguishing infectious lung lesions from malignancy and emphasizes the role of metagenomic next-generation sequencing (mNGS) in identifying rare anaerobic pathogens, offering valuable insights into accurate diagnosis and tailored treatment approaches.

## Case presentation

### Patient information and initial presentation

A 79-year-old man with a medical history of hypertension, well controlled with regular antihypertensive medication, and no other significant comorbidities, was admitted with a one-week history of persistent cough and sputum production. He also reported occasional dyspnea and subjective fever, although no body temperature was documented. He had a 25 pack-year smoking history (10 cigarettes per day for 50 years) and consumed alcohol occasionally in small amounts. He denied hemoptysis, chest pain, recent travel, night sweats. A recent decline in appetite was noted.

### Initial assessment and diagnostic workup

**Figure 1:**
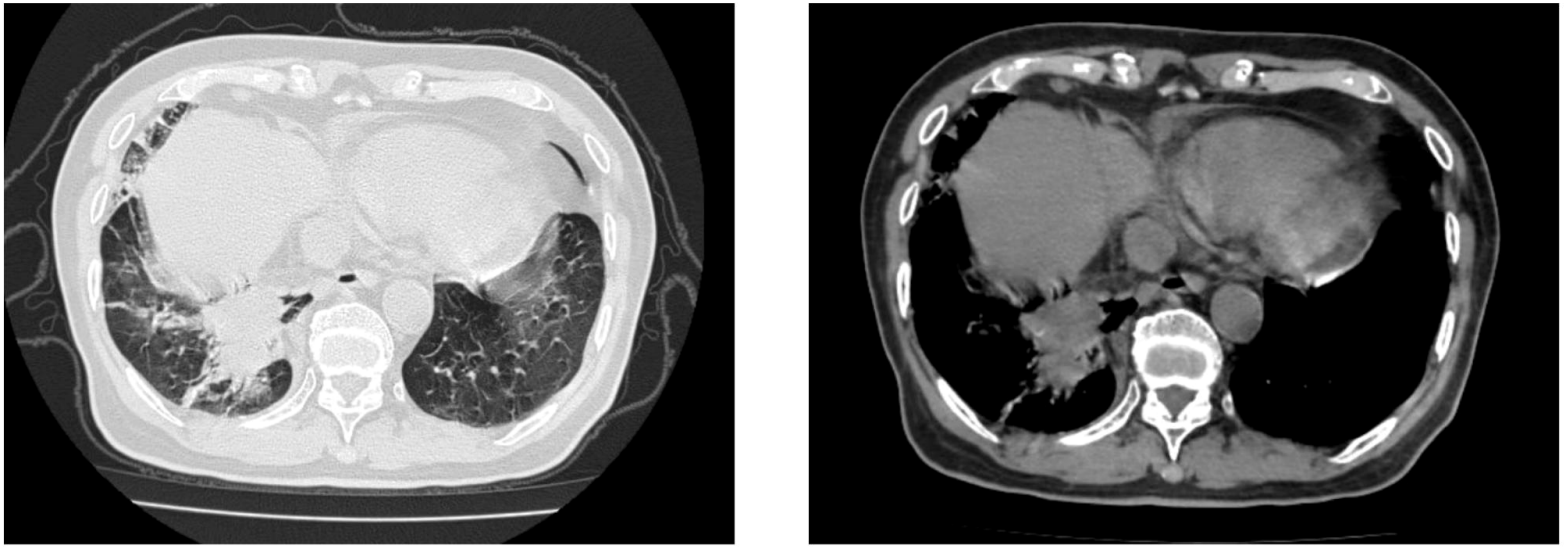
Chest computed tomography (CT) scan revealed a lobulated mass in the posterior basal segment of the right lower lobe, accompanied by partial subsegmental bronchial obstruction and distal inflammatory changes. Additionally, an increased soft tissue density was observed in the right lower hilar region.

**Figure 2:**
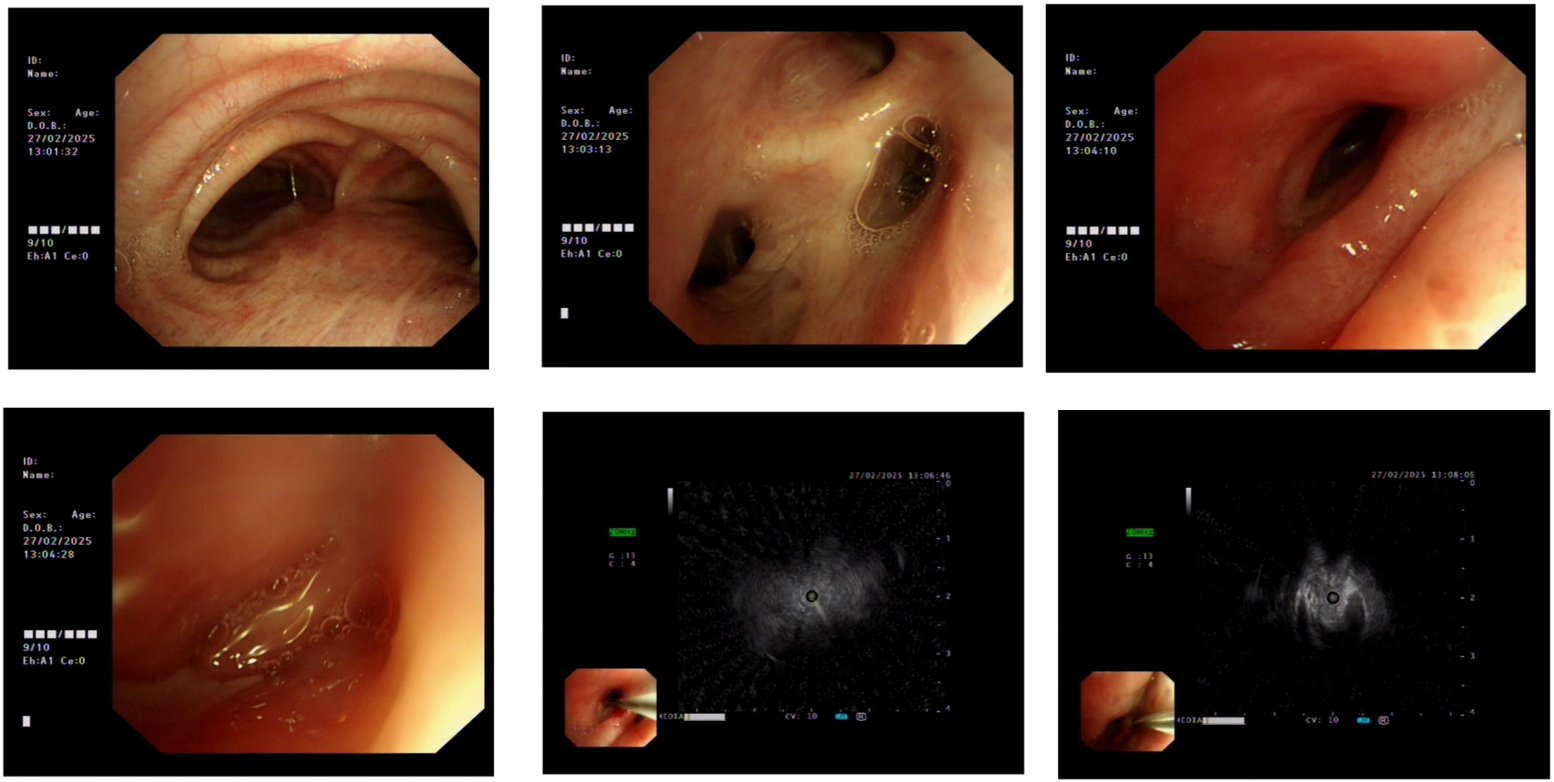
External compression-induced stenosis was observed at the bronchial openings of the right middle and lower lobes. Endobronchial ultrasound (EBUS) and brush biopsy were performed for further evaluation.

**Figure 3:**
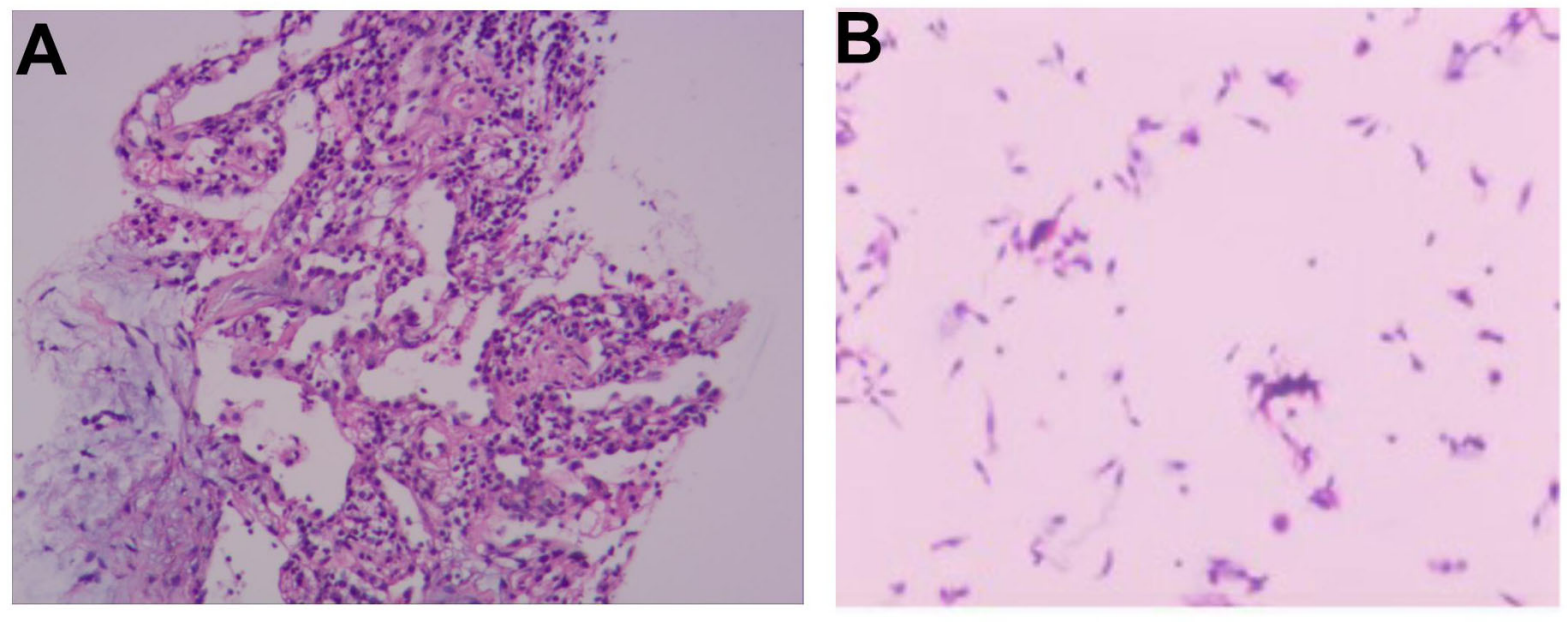
**(A)** Histopathological analysis of the EBUS-guided lung biopsy shows abundant ciliated columnar epithelial cells and inflammatory infiltrates, with no evidence of malignant cells. **(B)** Bronchial brush smear reveals numerous ciliated columnar epithelial cells and inflammatory cells.

At the time of admission, laboratory tests revealed markedly elevated inflammatory markers (**Table 1**), while routine hematological and biochemical parameters remained within normal limits. Chest computed tomography (CT) showed a lobulated mass in the posterior basal segment of the right lower lobe, accompanied by partial subsegmental bronchial obstruction and distal inflammatory changes. Additionally, increased soft tissue density was observed in the right lower hilar region, along with mediastinal lymphadenopathy (**Figure 1**). Given the patient’s advanced age and long-standing history of heavy smoking, the radiological findings were highly suspicious for malignancy. A preliminary diagnosis of malignant tumor with obstructive pneumonia was made. To further evaluate the lesion, an endobronchial ultrasound-guided biopsy (EBUS) was performed, and tissue specimens were submitted for histopathological examination (**Figure 2**). However, the pathology revealed abundant ciliated columnar epithelial cells and inflammatory infiltration (**Figure 3**), with no evidence of malignant cells.

**Table 1:** Laboratory.

Item	Actual value	Normal value
Complete blood count		
Day1		
WBC	11.43 × 10^9^/L	(3.50–9.50) × 10^9^/L
Neutrophils	10.37 × 10^9^/L	(1.80–6.30) × 10^9^/L
Lymphocyte	0.74 × 10^9^/L	(1.10–3.20) × 10^9^/L
CRP	199.9mg/L	(0–3) mg/L
PCT	0.189ng/ml	(0–0.052) ng/mL
Day4		
WBC	6.41 × 10^9^/L	(3.50–9.50) × 10^9^/L
Neutrophils	5.95 × 10^9^/L	(1.80–6.30) × 10^9^/L
Lymphocyte	0.32 × 10^9^/L	(1.10–3.20) × 10^9^/L
CRP	260.3mg/L	(0–3) mg/L
PCT	5.400ng/ml	(0–0.052) ng/mL
IL-6	412.40pg/ml	(0–6.6) pg/mL
Day7		
WBC	8.59 × 10^9^/L	(3.50–9.50) × 10^9^/L
Neutrophils	7.65 × 10^9^/L	(1.80–6.30) × 10^9^/L
Lymphocyte	0.63 × 10^9^/L	(1.10–3.20) × 10^9^/L
CRP	180.9mg/L	(0–3) mg/L
PCT	0.938ng/ml	(0–0.052) ng/mL
IL-6	137.10pg/ml	(0–6.6) pg/mL
Day10		
WBC	12.40 × 10^9^/L	(3.50–9.50) × 10^9^/L
Neutrophils	11.04 × 10^9^/L	(1.80–6.30) × 10^9^/L
Lymphocyte	1.06 × 10^9^/L	(1.10–3.20) × 10^9^/L
CRP	124.0mg/L	(0–3) mg/L
PCT	0.275ng/ml	(0–0.052) ng/mL
IL-6	81.30pg/ml	(0–6.6) pg/Ml
Day16		
WBC	9.74 × 10^9^/L	(3.50–9.50) × 10^9^/L
Neutrophils	9.13 × 10^9^/L	(1.80–6.30) × 10^9^/L
Lymphocyte	0.48 × 10^9^/L	(1.10–3.20) × 10^9^/L
CRP	83.79mg/L	(0–3) mg/L
Day20		
WBC	4.37× 10^9^/L	(3.50–9.50) × 10^9^/L
Neutrophils	2.63× 10^9^/L	(1.80–6.30) × 10^9^/L
Lymphocyte	1.55 × 10^9^/L	(1.10–3.20) × 10^9^/L
CRP	6.7mg/L	(0–3) mg/L
Day27		
WBC	6.91× 10^9^/L	(3.50–9.50) × 10^9^/L
Neutrophils	5.05× 10^9^/L	(1.80–6.30) × 10^9^/L
Lymphocyte	1.56 × 10^9^/L	(1.10–3.20) × 10^9^/L
CRP	4.3mg/L	(0–3) mg/L

### Initial treatment and disease progression

**Figure 4:**
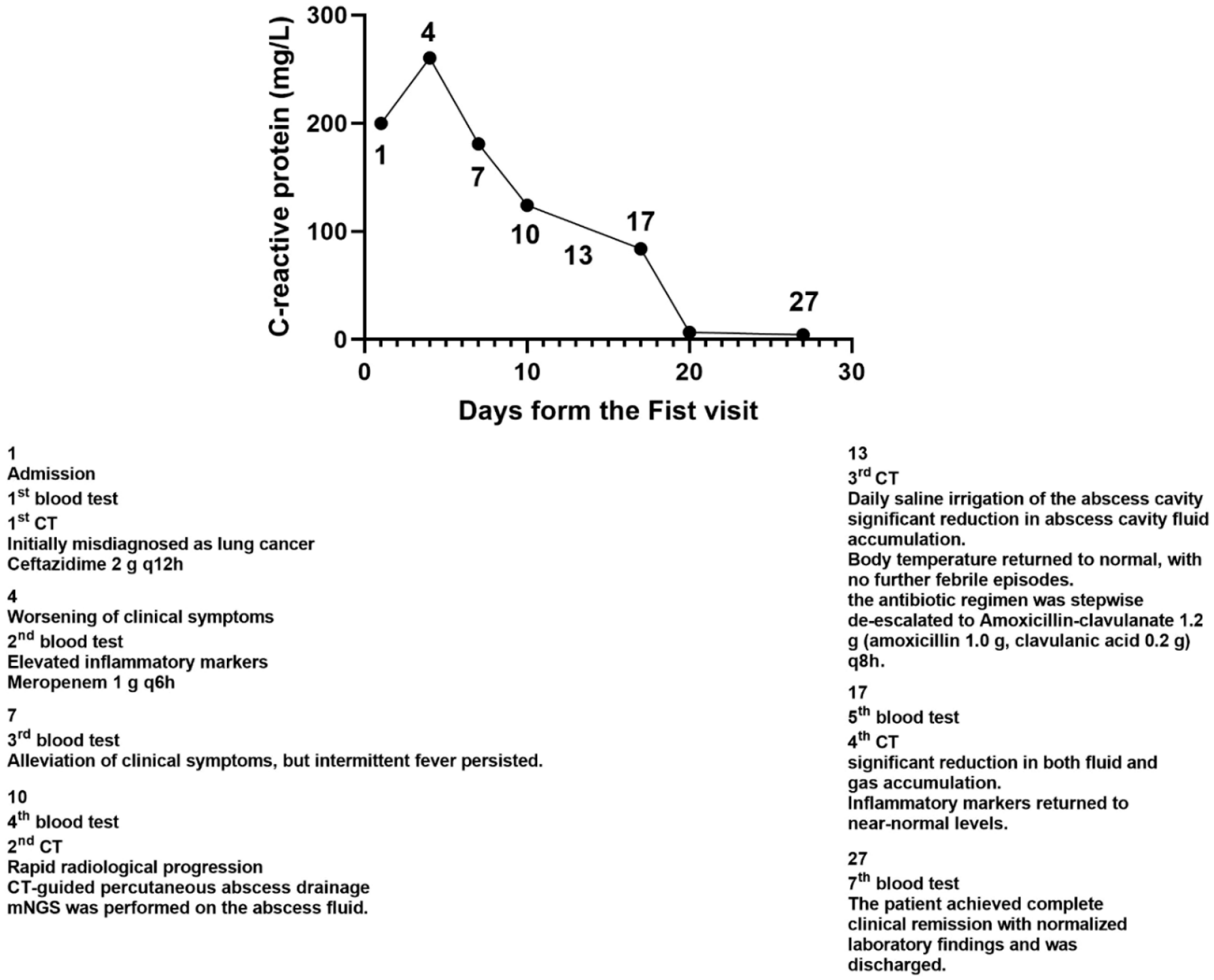
Clinical course and management.

The patient initially received empirical antibiotic therapy with ceftazidime (2 g every 12 hours for a total of 3 days). However, he continued to experience persistent high-grade fever, recurrent febrile episodes, worsening cough and sputum production, and progressively aggravated dyspnea. On day 4, repeat laboratory investigations revealed a worsening inflammatory response: white blood cell (WBC) count was 6.41 × 10^9^/L, neutrophil count was 5.95 × 10^9^/L (92.80%), C-reactive protein (CRP) was 260.3 mg/L, procalcitonin (PCT) was 5.400 ng/mL, and interleukin-6 (IL-6) was 412.40 pg/mL. In light of the clinical deterioration, the antibiotic regimen was escalated to meropenem (1 g every 6 hours), and blood samples were collected for culture prior to the initiation of the upgraded antimicrobial therapy. Following the escalation of antimicrobial therapy, the patient showed slight improvement in dyspnea and cough; however, intermittent fever persisted. The detailed treatment course is presented in **Figure 4**.

### Radiological progression and emergency intervention

**Figure 5:**
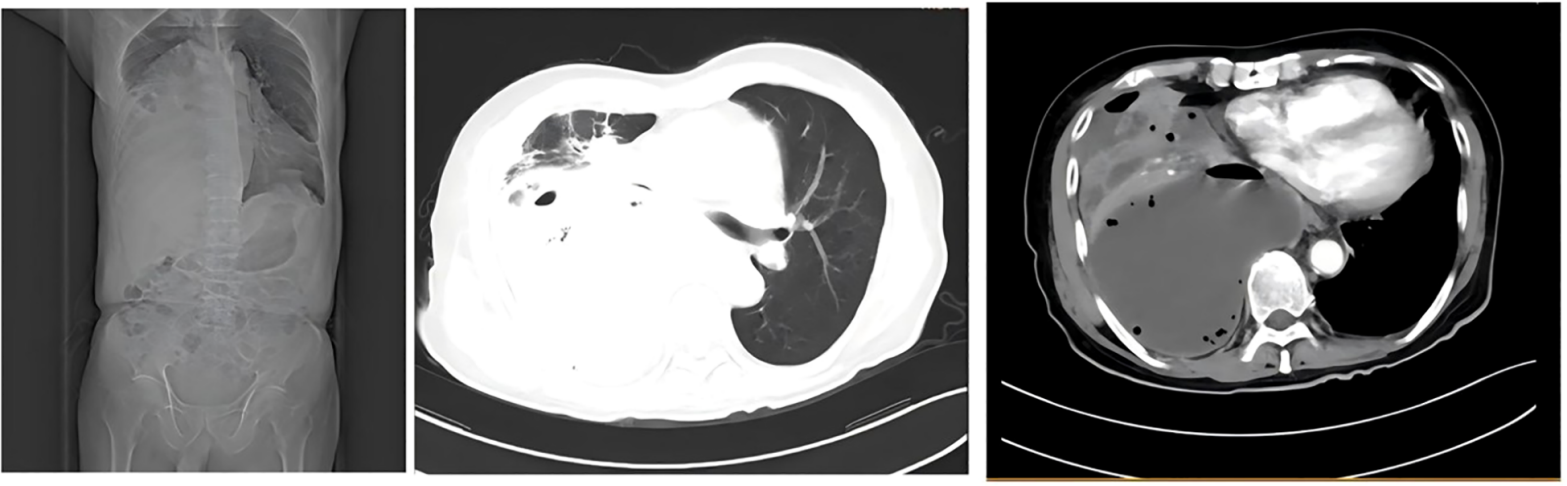
Severe compressive atelectasis of the right middle and lower lobes; anterior displacement of the pulmonary venous trunk and main bronchus; leftward mediastinal shift with cardiac silhouette deviation.

On day 10 after admission, contrast-enhanced chest CT revealed a rapidly enlarging fluid-density lesion, with multiple scattered gas collections in the right middle and lower lobes, measuring 135 × 110 mm (CT attenuation: 16 HU, non-contrast) (**Figure 5**). These findings were highly suggestive of progressive infective consolidation with abscess formation, leading to:

- Severe compressive atelectasis of the right middle and lower lobes
- Anterior displacement of the pulmonary venous trunks and main bronchi
- Leftward mediastinal shift with deviation of the cardiac silhouette

**Figure 6:**
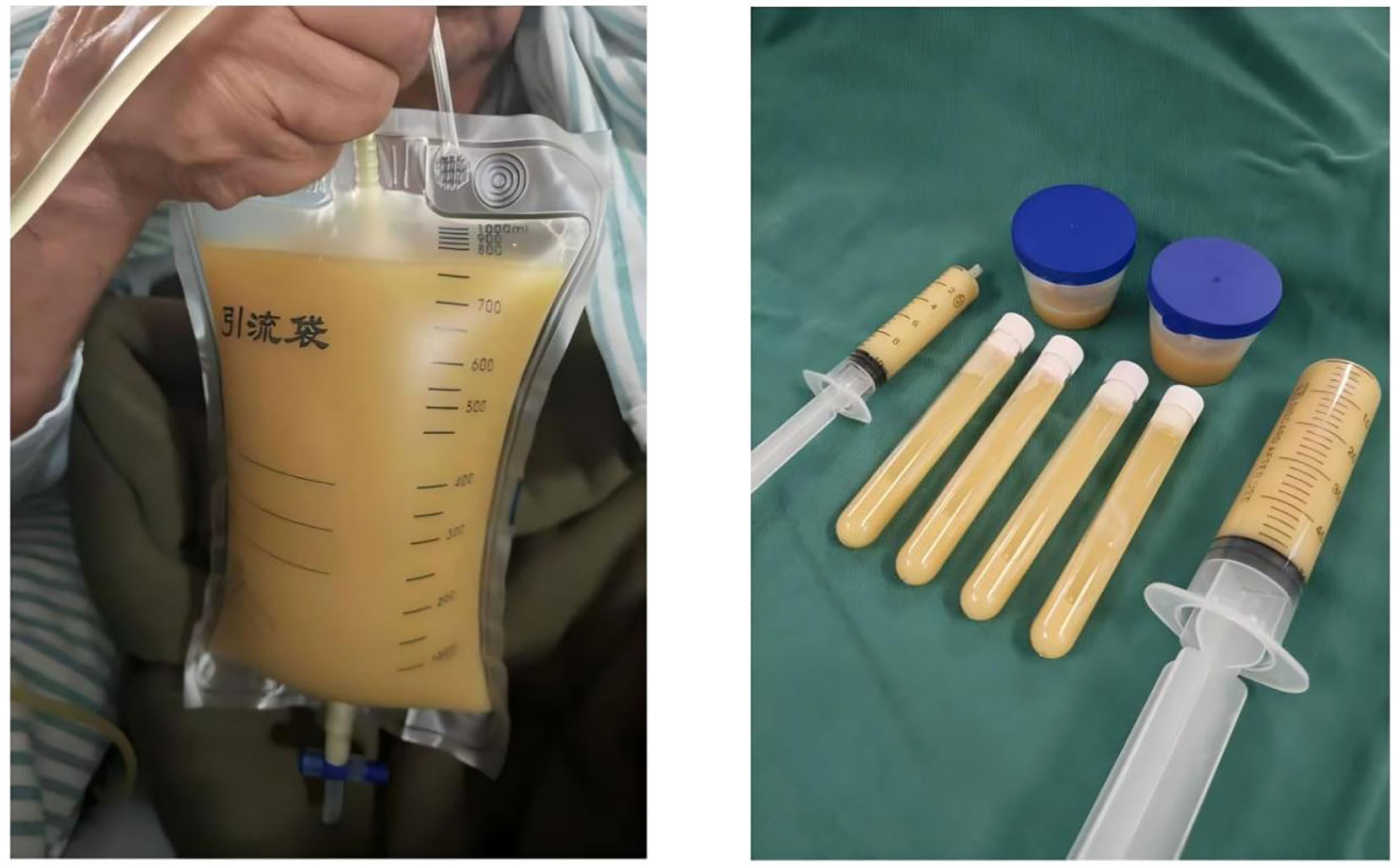
CT-guided percutaneous abscess drainage was performed, yielding a large volume of thick, purulent fluid.

Given the rapid disease progression within a short timeframe, an emergency CT-guided percutaneous abscess drainage was performed, yielding a substantial volume of thick, purulent fluid (**Figure 6**).

### Pathogen identification and clinical response to treatment

**Figure 7:**
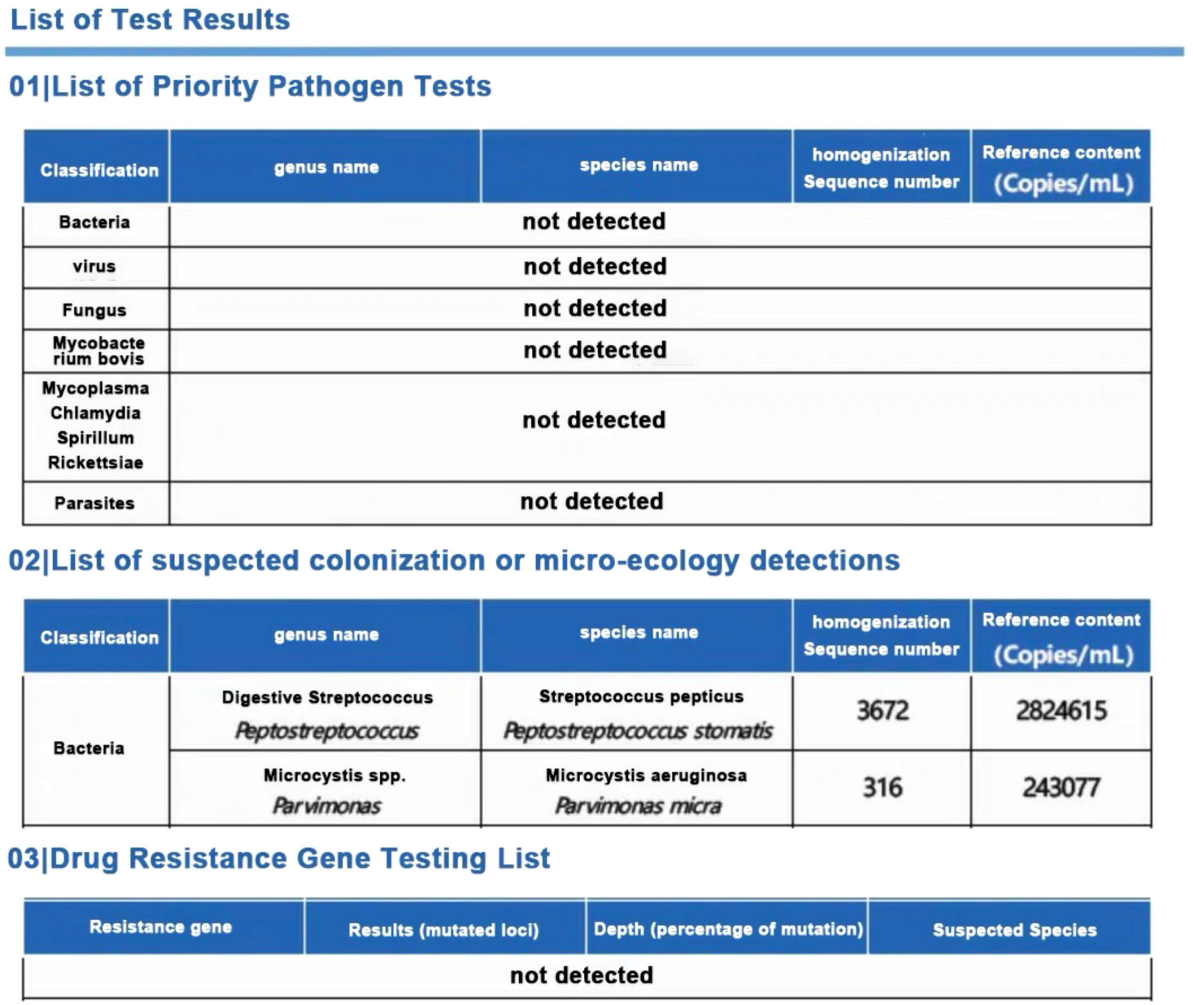
Original mNGS result image.

**Figure 8:**
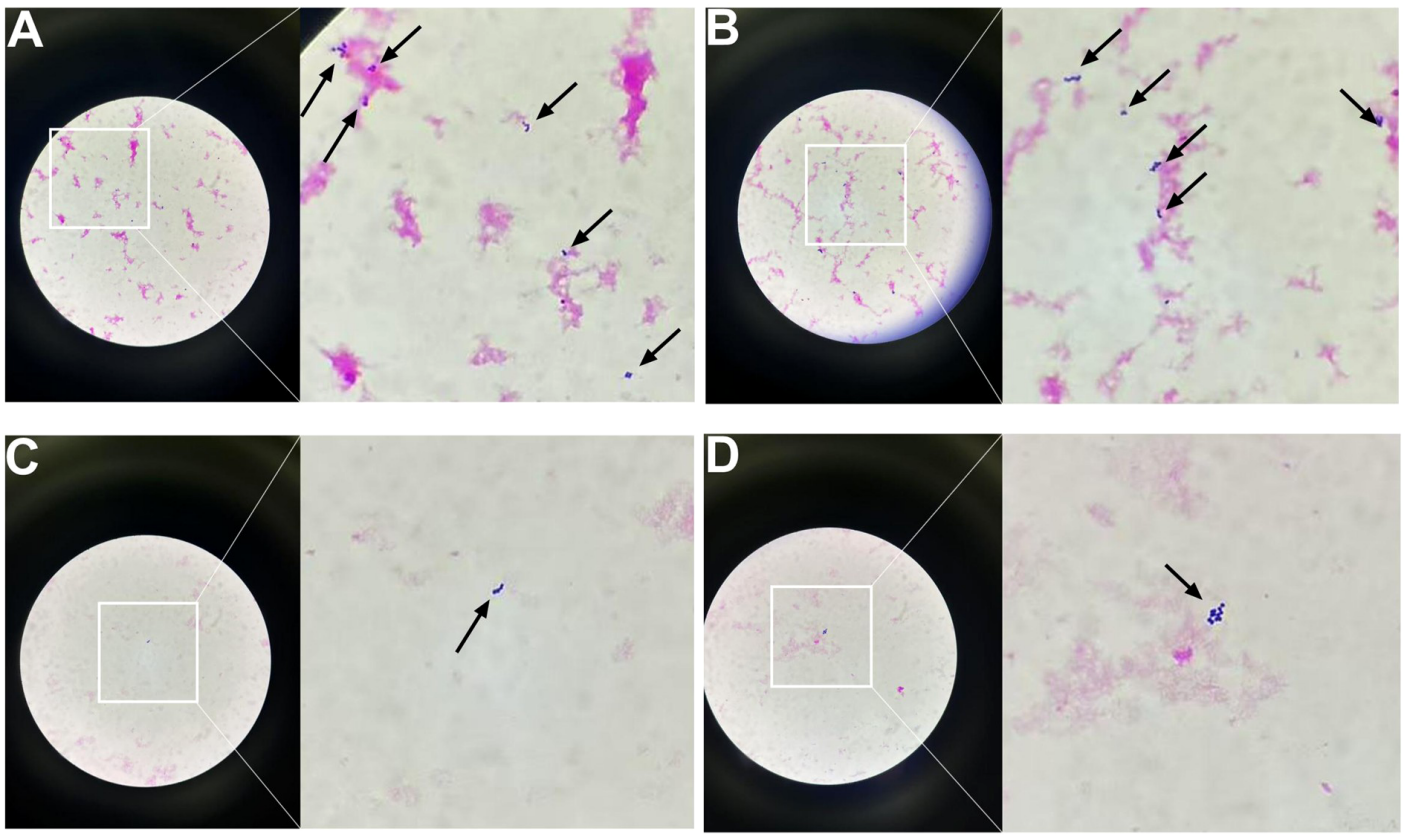
Gram-stained smears of positive blood culture showing Gram-positive cocci under light microscopy (original and magnified views). **(A)** Numerous Gram-positive cocci forming dense clusters and short chains. **(B)** Gram-positive cocci arranged predominantly in long, branching chains. **(C)** Sparse distribution of Gram-positive cocci, with isolated organisms visible. **(D)** Small clusters of Gram-positive cocci with moderate density.

To identify the causative pathogens, metagenomic next-generation sequencing (mNGS) was performed on the abscess fluid. The results exclusively detected *Peptostreptococcus stomatis* (Sequence ID: 3672) and *Parvimonas micra* (Sequence ID: 316) (**Figure 7**). Metagenomic sequencing was performed using the Nanopore TNGS platform, yielding 25 million bases and 37,874 high-quality reads, which exclusively identified *Peptostreptococcus stomatis* and *Parvimonas micra.* Given the presence of pleural empyema and gas formation, which are characteristic features of anaerobic infections, along with the blood culture microscopy findings showing Gram-positive bacteria arranged in chains or clusters (**Figure 8**), the diagnosis of *Peptostreptococcus stomatis* and *Parvimonas micra* infection was confirmed.

**Figure 9:**
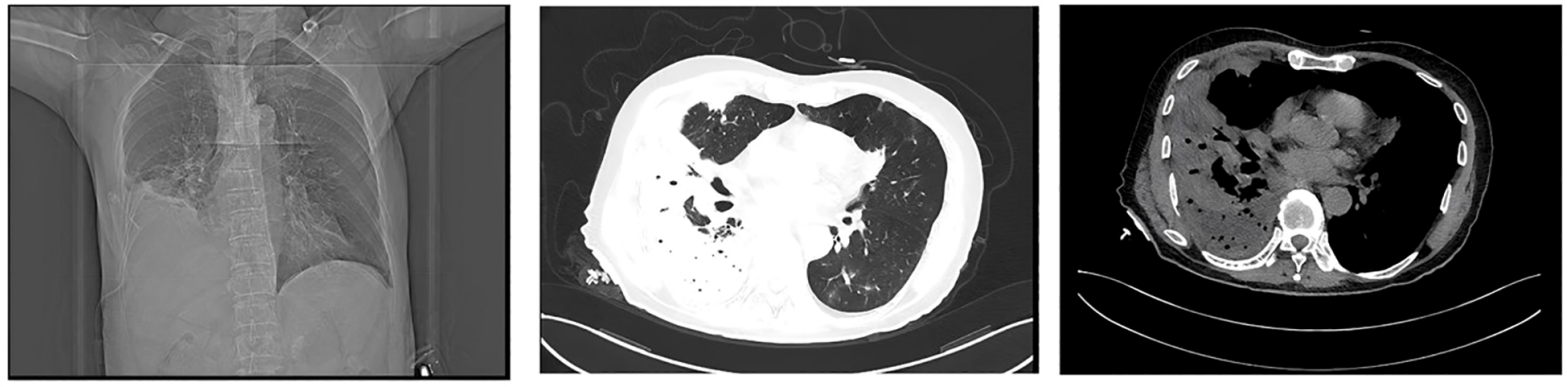
On post-procedure day 3, a follow-up chest CT demonstrated a significant reduction in abscess cavity fluid accumulation.

**Figure 10:**
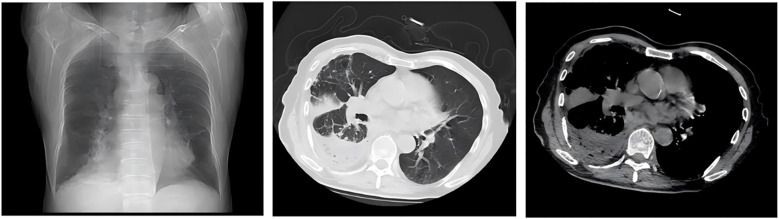
By post-procedure day 7, a repeat chest CT revealed a substantial decrease in both fluid and gas accumulation compared to prior imaging.

Following percutaneous abscess drainage, the patient’s condition steadily improved, with body temperature returning to normal. Over the subsequent days, his respiratory symptoms, including dyspnea, cough, and sputum production, gradually subsided. At the same time, inflammatory markers decreased, and vital signs remained stable, suggesting effective infection control. On post-procedure day 3, a follow-up chest CT demonstrated a significant reduction in abscess cavity fluid accumulation (**Figure 9**), indicating progressive infection resolution. In the subsequent days, daily saline irrigation of the abscess cavity was performed alongside intrapleural urokinase injections to enhance drainage. As the patient’s clinical condition continued to improve, the antibiotic regimen was stepwise de-escalated to Amoxicillin-clavulanate 1.2 g (amoxicillin 1.0 g, clavulanic acid 0.2 g) q8h. By post-procedure day 7, a repeat chest CT (**Figure 10**) demonstrated a significant reduction in both fluid and gas accumulation compared to previous imaging, indicating continued resolution of the infection. Laboratory findings showed a stabilized inflammatory response, with a white blood cell (WBC) count of 4.37 × 10^9^/L, a neutrophil count of 2.63 × 10^9^/L (60.20%), and a C-reactive protein (CRP) level of 6.7 mg/L.

At discharge, the patient had no fever, was breathing comfortably, and could walk without experiencing respiratory distress, indicating that the infection had been effectively controlled and his condition had stabilized. A follow-up plan was arranged to monitor his recovery and assess long-term treatment outcomes.

## Discussion

This case report describes a rapidly progressive pulmonary abscess caused by *Peptostreptococcus stomatis* and *Parvimonas* micra, two strict anaerobes that have not previously been reported as primary pathogens in invasive pulmonary infections in immunocompetent individuals. The rapid disease progression and radiological resemblance to lung cancer in this patient highlight the critical need for timely differentiation between infectious lung lesions and malignancy to prevent misdiagnosis and ensure appropriate treatment.

### Diagnostic challenges and the role of mNGS

In this case, the radiological findings closely resembled those of lung cancer, leading to an initial misdiagnosis and highlighting the challenges in differentiating between malignant and infectious lung lesions based on imaging alone. Conventional diagnostic methods, including CT scans and histopathological examination of bronchoscopic biopsy samples, failed to provide a definitive diagnosis. The CT images revealed a lobulated mass with irregular margins and central necrosis, features highly suggestive of malignancy, which initially guided clinicians toward a neoplastic diagnosis. This case underscores the limitations of relying solely on traditional imaging and histopathology for distinguishing between infectious and malignant lung lesions, particularly in the absence of characteristic clinical features. It further emphasizes the need for advanced molecular diagnostic techniques in complex cases. In this case, metagenomic next-generation sequencing (mNGS) played a crucial role in pathogen identification. Traditional culture methods have limitations in detecting anaerobic bacteria, particularly *Peptostreptococcus stomatis* and *Parvimonas micra*, which are slow-growing and require specific culture conditions. These characteristics often make them difficult to identify using conventional diagnostic techniques. As a culture-independent, high-throughput sequencing technology, mNGS successfully detected *Peptostreptococcus stomatis* and *Parvimonas micra*, confirming the diagnosis of anaerobic infection. Additionally, the presence of pleural empyema and gas formation in imaging findings further supported an anaerobic infectious etiology. The broad-spectrum detection capability of mNGS allows for comprehensive pathogen identification, which is particularly beneficial in cases involving fastidious anaerobes, patients who have received empirical antibiotic therapy prior to sample collection, or complex infections where conventional tests fail to identify the causative agent (Chiu and Miller, 2019). The successful management of this case further supports the clinical utility of mNGS in diagnosing rare or atypical infections and underscores its potential role in guiding treatment strategies for complex infectious diseases.

### Pathogen analysis: *Peptostreptococcus stomatis* and *Parvimonas micra*

*Peptostreptococcus* species are anaerobic, non-spore-forming, gram-positive cocci that are part of the normal microbial flora of the oral cavity, upper respiratory tract, gastrointestinal tract, female genitourinary system, and skin (Murphy and Frick, 2013). These bacteria are known to cause various deep-seated infections, including skin and soft tissue abscesses, periodontitis, aspiration pneumonia, and intra-abdominal infections (Murphy and Frick, 2013). Additionally, they have been implicated in bloodstream infections (Brook, 2002), perirenal abscesses (Hasel et al., 2022), infective endocarditis (Verdecia et al., 2019), and prostatic abscesses (Barnes et al., 2020). Similarly, *Parvimonas micra*, a member of the *Parvimonas* genus, is a facultative anaerobic, gram-positive coccus (0.3–0.7 µm) commonly found as part of the normal flora in the oral cavity, upper respiratory tract, and gastrointestinal system (Murphy and Frick, 2013). Although generally commensal, *Parvimonas micra* can act as an opportunistic pathogen, particularly in immunocompromised individuals, leading to severe infections such as pulmonary abscesses (Fukushima et al., 2023), spinal epidural abscesses (Yang et al., 2023), and psoas muscle abscesses (Yoo et al., 2019). Identification of *Parvimonas micra* is often challenging due to its slow growth, lack of distinctive clinical symptoms, and the requirement for specialized culture media and identification techniques (Roy et al., 2017; Sultan et al., 2018).

Although anaerobic infections are generally considered to follow an indolent clinical course, the rapid progression observed in this patient suggests the involvement of additional pathogenic mechanisms. Potential contributing factors may include a high bacterial load, upregulated expression of virulence factors, or localized immune dysregulation, despite the patient being immunocompetent. Notably, *Parvimonas micra* and *Peptostreptococcus stomatis* are members of the oral microbiota and possess significant suppurative potential. In retrospect, an important clinical detail—pre-admission dental pain—was initially overlooked by both the medical team and the patient. The identification of oral anaerobes as the predominant pathogens, coupled with this dental history, strongly supports the hypothesis that the pulmonary abscess originated from aspiration pneumonia.

This hypothesis is further substantiated by anatomical and epidemiological considerations. The lesion was located in the right lower lobe—a common site for aspiration-related infections due to the more vertical orientation of the right main bronchus—particularly in elderly individuals. These features are consistent with well-established epidemiological data indicating that a substantial proportion of pulmonary abscesses are attributable to unrecognized aspiration events.

Despite the limited epidemiological data available on *P. micra* and *P. stomatis*, the widespread adoption of metagenomic next-generation sequencing (mNGS) has significantly improved the detection of anaerobic pathogens. Emerging evidence suggests that these organisms may be more prevalent in severe pulmonary and systemic infections than previously appreciated.

### Treatment strategy and clinical significance

The standard treatment for lung abscesses caused by anaerobic bacteria involves prolonged antibiotic therapy with appropriate anaerobic coverage. Clindamycin (600 mg IV every 8 hours) has demonstrated superior efficacy compared to penicillin in multiple clinical trials, showing higher response rates, shorter fever duration, and faster resolution of putrid sputum (Bartlett, 2013). Recommended antibiotic regimens for lung abscesses include β-lactam antibiotics combined with β-lactamase inhibitors (such as ticarcillin-clavulanate, ampicillin-sulbactam, amoxicillin-clavulanate, and piperacillin-tazobactam), chloramphenicol, imipenem or meropenem, second-generation cephalosporins (cefoxitin, cefotetan) (Kuhajda et al., 2015), and newer fluoroquinolones like moxifloxacin, which has been shown to be as effective as ampicillin-sulbactam (Ott et al., 2008).

For abscesses exceeding 6 cm in diameter or cases where symptoms persist beyond 12 weeks despite appropriate therapy, conservative treatment alone is often insufficient. If the patient’s general condition permits, surgical intervention should be considered. Surgical options include chest tube drainage or resection of the lung abscess along with the surrounding affected tissue (Kuhajda et al., 2015).

In this case, the patient was initially treated with ceftazidime; however, due to its limited activity against anaerobic pathogens, infection control was inadequate. As the clinical condition deteriorated, antimicrobial therapy was escalated to meropenem, which offers broad-spectrum coverage against anaerobes. Although the patient showed partial symptomatic relief, intermittent fever persisted, and follow-up imaging revealed rapid enlargement of the abscess, accompanied by compressive atelectasis of the right lung and leftward mediastinal shift—findings indicative of a large lesion exerting significant mass effect. Consequently, emergency CT-guided percutaneous abscess drainage was performed.

Following combined treatment with meropenem and percutaneous drainage, the patient experienced marked clinical improvement, including normalization of body temperature, resolution of dyspnea and cough, and a significant decline in inflammatory markers. These outcomes strongly support the effectiveness of the combined therapeutic strategy in this case.

## Conclusion

This case highlights the importance of accurately distinguishing between malignant pulmonary lesions and infectious lung abscesses to prevent unnecessary invasive procedures and ensure timely and appropriate treatment. It also presents, for the first time, *Peptostreptococcus stomatis* and *Parvimonas micra* as causative agents of rapidly progressive pulmonary abscesses, broadening our understanding of the role of anaerobic bacteria in lung infections. Moreover, this case underscores the value of metagenomic next-generation sequencing (mNGS) in identifying rare pathogens in complex infections. When faced with rapidly progressing pulmonary lesions, clinicians should remain vigilant for the possibility of anaerobic infections and consider early interventional treatment to improve patient outcomes.

As a clinical reflection, we note that the initial EBUS biopsy specimen was not sent for microbiological culture due to a preliminary suspicion of pulmonary malignancy. In hindsight, this step proved to be critically important. We have included this reflection to remind clinicians of the necessity of comprehensive microbiological assessment when the diagnosis is uncertain.

## Data Availability

The original contributions presented in the study are included in the article/Supplementary Material. Further inquiries can be directed to the corresponding authors.
